# Genetic testing for exercise prescription and injury prevention: AIS-Athlome consortium-FIMS joint statement

**DOI:** 10.1186/s12864-017-4185-5

**Published:** 2017-11-14

**Authors:** Nicole Vlahovich, David C. Hughes, Lyn R. Griffiths, Guan Wang, Yannis P. Pitsiladis, Fabio Pigozzi, Nobert Bachl, Nir Eynon

**Affiliations:** 1 Australian Institute of Sport (AIS), Australian Sports Commission, Canberra, Australia; 2 Genomics Research Centre, Institute of Health and Biomedical Innovation (IHBI), Queensland University of Technology, Brisbane, Australia; 3 Reference Collaborating Centre of Sports Medicine for Anti-Doping Research, University of Brighton, Eastbourne, UK; 4 Department of Movement Human and Health Sciences University of Rome “Foro Italico”, Rome, Italy; 5 International Federation of Sports Medicine (FIMS), Lausanne, Switzerland; 6 Department of Sports and Exercise Physiology, Centre for Sports Science and University Sports of the University of Vienna, Vienna, Austria; 7 Institute of Sport, Exercise and Active Living (ISEAL), College of Sport and Exercise Science, Victoria University, PO Box 14428, Melbourne, VIC 8001 Australia; 8 University of Canberra Research Institute for Sport and Exercise (UCRISE), University of Canberra, Canberra, Australia

## Abstract

**Background:**

There has been considerable growth in basic knowledge and understanding of how genes are influencing response to exercise training and predisposition to injuries and chronic diseases. On the basis of this knowledge, clinical genetic tests may in the future allow the personalisation and optimisation of physical activity, thus providing an avenue for increased efficiency of exercise prescription for health and disease.

**Results:**

This review provides an overview of the current status of genetic testing for the purposes of exercise prescription and injury prevention. As such there are a variety of potential uses for genetic testing, including identification of risks associated with participation in sport and understanding individual response to particular types of exercise. However, there are many challenges remaining before genetic testing has evidence-based practical applications; including adoption of international standards for genomics research, as well as resistance against the agendas driven by direct-to-consumer genetic testing companies. Here we propose a way forward to develop an evidence-based approach to support genetic testing for exercise prescription and injury prevention.

**Conclusion:**

Based on current knowledge, there is no current clinical application for genetic testing in the area of exercise prescription and injury prevention, however the necessary steps are outlined for the development of evidence-based clinical applications involving genetic testing.

## Background

Physical inactivity accounts for approximately 6% of the worldwide burden of disease and globally, around 23% of adults aged 18 years and over did not meet physical activity guidelines in 2010 [1]. Physical inactivity increases the risk of developing a range of conditions including hypertension, coronary heart disease, stroke, diabetes, breast and colon cancer; while physical activity is a key determinant of energy expenditure, and fundamental to energy balance and weight control [1]. Engaging in physical activity on a regular basis (e.g., exercise training) is therefore crucial for increasing cardiorespiratory fitness and decreasing the risk for chronic diseases. Physical activity levels and the response to similar exercise training vary considerably, with some people being ‘low/medium responders’ (with limited improvements following exercise training), while others respond well or very well (‘high responders’) [2]. This variable response appears to be influenced by both environmental (e.g. training status, nutrition, social economic status) and genetic factors.

While there have been some advances in knowledge and understanding of how genes are influencing the response to exercise training and predisposition to injuries and chronic diseases, the development of genetic tests which allow the personalisation and optimisation of physical activity remains elusive [3]. In the future, such tests may provide an avenue for increased efficiency of exercise prescription and injury prevention. Additionally, there are a range of genetic conditions that may impede an individual’s participation in exercise, with the most severe of these leading to an increased risk of sudden cardiac death [4, 5]. In particular, genes associated with cardiomyopathies may be of interest to sports medicine physicians [5]. Discovering genes that are associated with the predisposition of the severest of common sports injuries has become a particular focus of research interest.

Minimising time loss from injury has been correlated with athletic success for both teams and individuals [6, 7]. Additionally, sports injuries in recreational athletes have significant public health impact and consequences for future participation in sport and recreational activities [8]. Comprehensive injury surveillance is performed by many professional sporting bodies to examine the impact of particular injuries. The international bodies such as the International Olympic Committee (IOC) and the Fédération Internationale de Football Association (FIFA) systematically survey injuries in their major events [9–12]. For example, analysis of the London Olympic Games found that 11% of athletes reported an injury during the Games [12]. Prevention of injury in the sports arena is a high priority for athletes, coaches, high performance staff and medical personnel. Consequently, there has been significant research in the area of genetic variation and its impact on susceptibility to exercise-related injuries, with a particular focus on tendon and ligament injuries [13]. Much of this research is aimed at determining an individual’s susceptibility to, and/or risk of acquiring a sport injury and implementing preventative strategies.

The rapid development of genetic and genomic techniques has led to an increase in interest in the genetics of physical activity and sport. The Athlome Project Consortium was established in 2015 to collectively study the limited genotype and phenotype data available in elite athletes, as well as focusing on adaptation to exercise training and exercise-related musculoskeletal injuries [14]. The ultimate goal of this consortium is to inform personalised training and injury prevention, as well as informing doping detection utilising collaborative and rigorous research. Often studies within the field of exercise/injury genomics are limited by cohort sizes and other methodological concerns. The Athlome consortium has recently advocated an approach to overcome the main gaps in this research field [14], and to serve as a collective guiding reference in the identification of reliable genetic tests for exercise training and sports injuries. This review provides a statement on the state of play in genetic testing for the purposes of exercise prescription and injury prevention.

## Potential uses of genetic testing for exercise and health

### Identifying those who are at increased risk (or resilient) to musculoskeletal injury

Participation in sport or exercise training can lead to acute and chronic musculoskeletal injuries. For athletes, time lost from training and competition due to injury has a profoundly negative impact on performance. Additionally, when a member of the public who is engaged in exercise training acquires an injury this can lead to a lack of motivation towards, or fear of returning to training [15]. Several factors, both intrinsic and extrinsic, are reported to predispose an individual to musculoskeletal injury, including demographic factors (e.g., sex, age, weight, and height), anatomical factors (e.g., leg length discrepancy, malalignment and decreased flexibility), and environmental conditions (e.g., training patterns, technique flaws and equipment) [16–19]. Genetic variations also play a role in the risk profile for musculoskeletal injury [13].

Identification of gene variations that may lead to an increased or decreased risk of sport/exercise injury has been studied for a range of conditions including ligament [20–23], tendon [22, 24–27], muscle [28, 29], and bone injuries [30, 31]. Primarily this has been done using the hypothesis-driven, candidate gene approach, where a case-control cohort is examined for genetic variation in genes of interest and the occurrence of musculoskeletal injuries, focusing on genes involved in the extracellular matrix and apoptosis pathways (e.g., [13]). To date there has been only one genome-wide association study (GWAS) published in the area of sports injury, which did not identify any significant gene variations contributing to Achilles tendon or ACL injury [32]. The sample sizes of the candidate gene studies have been quite small (typically between 100 and 200 cases) [33]. In contrast, GWAS have utilised tens to hundreds of thousands of individuals to identify genetic variants in migraine and Alzheimer’s disease [34, 35]. It has been acknowledged that further investigation with appropriately sized cohorts is required to replicate and correctly interpret the association of identified polymorphisms with specific injuries [33]. Such studies should also be repeated in other populations, including non-Caucasian populations [26]. Importantly, gene-discovery studies should be accompanied by functional studies that would demonstrate, in either animal or human models, how newly identified gene variations could cause molecular/cellular changes leading to increased/decreased susceptibility to an injury.

There is future potential to use genetic screening in assessing risk of musculoskeletal injuries, providing an avenue to modify training, conditioning programs and physical therapy intervention in order to prevent injuries. The current level of evidence however does not support the clinical use of genetic screening. Further research is required to gain a deeper understanding of the range of gene variants that contribute to risk of injury and the effectiveness of personalised training regimens in reducing injury incidence when compared to usual training.

### Identifying high/low responders to specific training protocols in healthy and diseased populations

Specific training protocols assist athletes, or exercise participants, in achieving fitness goals. Tailoring exercise programs to fit the specific needs of athletes and/or the general population may provide an efficient mechanism for rapid development of aerobic fitness and strength. From the response to exercise training literature, it has become clear that there is considerable individual variability in the response to similar exercise training [36]. The implications are that some individuals are ‘low responders’ (improving their fitness only slightly following a specific exercise training) while others respond well or very well (‘high responders’). Response to training also seems to depend on the type and length of the exercise training protocol. For example, intense intermittent exercise, or interval training, has gained popularity in the last few years and is a powerful stimulus to induce many of the physiological adaptations typically associated with traditional, moderate-intensity continuous training [37]. However, not everyone responds similarly to this type of training, and it would appear that genetic variants play an important role in this variable response [2]. Uncovering the genes behind the individual response to exercise training therefore has exciting implications for “personal medicine” and the development of individualised exercise training and health programs. This development could have important health and economic ramifications by ensuring that specific types of exercise interventions are prioritised to those most likely to attain the greatest benefit.

Several types of chronic diseases, including cancer, have been shown to benefit from generalised physical activity, such as walking, or exercise training [38–42]. Examples of the application of different training types have shown that some chronic conditions are better suited to a particular type of training. Studies have shown that resistance training can improve cognitive function in patients with mild cognitive impairment, and can increase muscle strength and fat-free mass in frail elderly patients with sarcopenia [43, 44]. Other studies have shown potential benefits to using either endurance or resistance training in chronic obstructive pulmonary disease, or that exercise and generalised physical activity is beneficial to those undergoing cancer treatment [45, 46]. The application of genomic techniques to develop individual exercise prescription based on the type of disease and the patient’s predicted response to exercise would assist in targeting treatment; so-called personalised medicine. While there is a great deal of research being undertaken to identify genetic predictors of response or non-response to specific exercise regimes, there are currently no valid genetic tests that can be clinically applied for this purpose.

### Identifying those who may have uncommon disorders and be at specific risk in sport

Some genetic disorders do confer a significant health risk for individuals undertaking strenuous activity. Sudden cardiac death is the leading medical cause of death in athletes, with variable incidence rate in athlete subgroups, with the highest risk being reported in male African-American/black athletes and basketball players [47]. Several gene variants have been shown to be associated with cardiac electrophysiology, arrhythmias, and sudden cardiac death, however very few of these studies have been replicated and functional implications of the genes are not always clear [4]. There is therefore a pressing need to conduct research that could enable the risk of sudden cardiac death for each individual to be established and determine if the characteristics of a particular sport increases that risk [48]. Athletes with diagnosed heart conditions, such as hypertrophic cardiomyopathy, may be advised against participation in competitive sports and discouraged from intense physical activity, dependent on the severity of their condition [49].

Marfan syndrome is an inherited connective tissue disorder associated with ocular, musculoskeletal and cardiovascular manifestations, characterised by a tall and slender build and disproportionately long limbs, posing potential lethal threat during high-intensity exercise [50]. Marfan syndrome has an incidence of 4–20/100,000 depending on the population studied and diagnostic criteria used [51]. Marfan syndrome results most commonly from mutations in the fibrillin-1 gene on chromosome 15, which encodes for the glycoprotein fibrillin [52]. Where a family history, symptom history, physical examination or diagnostic investigations (slit lamp ocular examination, echocardiogram) raise suspicion of Marfan syndrome, molecular studies of the fibrillin gene may be useful in clarifying the diagnosis. Marfan syndrome is one condition that disproportionately affects athletes in sports where height provides a distinct advantage due to the athletic phenotype caused by genetic mutations. Sudden death by aortic aneurysm and dissections represent the most serious clinical manifestation of this disease, and as such, sporting organisations where a tall slender build with long limbs is advantageous (such as volleyball and basketball) may see the need to mitigate this risk by using screening of athletes.

Conducting a clinical evaluation should always be the first step before performing a genetic test. Genetic testing is especially indicated in the following scenarios; positive family history of inherited heart disease (e.g., cardiomyopathies, channelopathies, aortopathies) or suspicion of that type of disease (e.g., syncope episodes, arrhythmias, cardiac arrest, sudden death); or when the athlete’s phenotype strongly indicates the presence of an inherited disease (Marfan syndrome) [49]. Sporting organisations wishing to conduct genetic investigations into conditions that may lead to increased risk of sudden cardiac death should engage a medical practitioner to ensure that appropriate clinical examination and counselling takes place prior to conducting genetic testing.

## Challenges for genetic testing in sport and exercise medicine

### Cohort size

One of the major limitations in the field of exercise/injury genomics is the relatively low sample size of subjects and/or the general population participating in exercise training studies. Major collaborative effort is required for the field to progess, and enhance our understanding of the genes that influence the response to exercise and predisposition to injury. Improving cohort numbers in current biobanks would pave the way for genome-wide testing. To date, the analysis of single variant candidate genes (often poorly justified) using low-throughput techniques has yielded conflicting findings and inconsistent results. Current GWAS and Whole-Genome Sequencing (WGS) technology means that millions of gene variants are analyzed per individual [53]. A recent study utilised 375,000 individuals to identify 38 loci related to susceptibility of migraine [35]. A similar style of genome-wide approach, designed with sufficient power could identify specific regions or variants that contribute to increased/decreased susceptibility to injuries and the response to exercise training.

As a result of the rapid advances in the development and uptake of high-throughput DNA-sequencing methods, progress is now being made in understanding the genetic basis of rare, and also some of the common diseases [54]. The first attempt to utilise a GWAS approach for athletic performance was recently undertaken by an international consortium (GAMES) [55]. This GWAS involved two cohorts of elite endurance athletes and controls (GENATHLETE and Japanese endurance runners), from which a panel of 45 promising markers was identified. These markers were tested for replication in seven additional cohorts of endurance athletes and controls: from Australia, Ethiopia, Japan, Kenya, Poland, Russia and Spain. This first of a kind study was based on a total of 1520 endurance athletes (835 who took part in endurance events in World Championships and/or Olympic Games) and 2760 controls. Although this investigation did not identify a panel of genomic variants common to these elite endurance athlete groups, some of the suggestive leads identified warrant further investigation in expanded comparisons of world-class endurance athletes and sedentary controls and in tightly controlled exercise training studies [55].

In a recent, more successful effort to discover the genes associated with muscle strength, Willems et al. [56] examined the genetic loci associated with hand grip strength, which is a marker of muscular fitness and frailty, with lower hand grip strength associated with lower quality of life. Results were obtained from a combined sample of 195,180 individuals, initially utilising a UK DNA Biobank, with follow up analysis in independent samples of elite sprinters. The significant cohort size lead to identification of 16 loci associated with grip strength (*P* < 5 × 10^−8^) in combined analyses. A number of these loci contain genes implicated in structure and function of skeletal muscle fibres (*ACTG1*), neuronal maintenance and signal transduction (*PEX14, TGFA, SYT1*), or monogenic syndromes with involvement of psychomotor impairment (*PEX14, LRPPRC* and *KANSL1*). Importantly, this recent discovery provides new biological insight into the mechanistic underpinnings of muscle strength.

### Cohort homogeneity

Another limitation common to the field of exercise/injury genomics is the cohort homogeneity. The majority of studies in sport and exercise genetics have been conducted using Caucasian/European subjects. While the homogeneity of the cohort assists in discovery of potential gene variants of significance, this poses a problem for the application of genetic tests to the broader community. Although we predict that the development of assessment tools will benefit from individual genomic information, not all of these applications may be extrapolated from the Caucasian cohorts to other populations. Encouragingly, there have been some recent attempts to study the genome of Asian (i.e., Chinese, Japanese, Taiwanese) and South American (i.e., Brazilian) athletes [57–59]. This trend is increasingly growing with the use of collaborative approaches and data sharing between international research centers.

An additional consideration in cohort homogeneity is the gene-by-sex interactions. Exercise genomic studies primarily utilise mixed cohorts of males and females, and account for sex differences as a covariate in their statistical analyses. For example, compelling findings of sex-dependent genetic effects on disease have been reported in type II diabetes [60] and autism spectrum disorders [61], which further complicates the issue of pooling together females and males into one cohort.

### Growth of DTC companies promoting non-evidence based testing

There has been a rapid expansion of direct-to-consumer (DTC) genetic testing services, with those services being provided to members of the public on a commercial basis without any involvement of a medical practitioner [62]. A range of companies offer DTC genetic testing, purporting to examine how one’s genes contribute to their athletic prowess. DTC companies commonly offer advice in terms of trainability or personalised training programs, predisposition to athletic success in power/endurance sports, and advice relating to weight loss management [62], despite the lack of evidence to support such advice. This type of DTC marketing targets athletes, parents, coaches and people from the general population seeking an athletic ‘edge’, and altered response to exercise training for success in sporting performance.

There are numerous problems inherent with the provision of advice based on the current repertoire of DTC genetic tests. In 2006, the United States Government Accountability Office (GAO) investigated companies selling DTC genetic tests and testified that these companies made medically unproven disease predictions [63]. For example, samples sent from the one individual to different DTC genetic companies resulted in inconsistent reporting between companies, including conflicting risk predictions for a range of diseases [63]. A systematic review of DTC genetic tests concluded that it is unacceptable that online companies offer genetic testing lacking scientific evidence and having no proven clinical utility, and make misleading marketing claims [64]. Recent research also suggests that DTC genetic testing companies massively exaggerate the predictive powers of their tests by distorting scientific evidence to support unfounded claims and in doing so have eroded the faith in the science behind genetic testing [65]. In response to this situation, a number of countries have instigated legislation to ensure that genetic testing cannot be carried out without the involvement of a medical practitioner, while there is no such legislated protection for consumers in certain other countries [66–68]. However, the DTC tests that draw conclusions about sporting performance do not provide health advice to the consumer and, therefore, may not meet the criteria to be regulated under current legislation.

A joint FIMS-Athlome Consensus Statement in 2015 warned against the use of DTC tests in athletes, stating that the current level of genetic knowledge is being misrepresented implicitly for commercial purposes and concluding that there is no place for DTC testing for predicting sports performance and talent identification [62]. Recently, the Australian Institute of Sport (AIS) has also developed a position statement to address the implications of recent advances in the field of genetics and the ramifications for the health and well-being of athletes [69].

### Potential ethical dilemmas

Numerous ethical dilemmas exist in relation to genetic testing, for purposes other than medicine. Genomic testing raises a number of important issues for those prescribing the test, including the complexity of informed consent, sample and data storage, return of results, testing involving children, and privacy and confidentiality [70]. The AIS detailed their position in relation to the ethical dilemmas facing genetic and genomic testing of athletes [69]. In brief, genomic and genetic testing for non-medical purposes must be well thought out, with clear and transparent planning for all aspects of information management outlined prior to the commencement of testing. The right of refusal for non-participation must be respected and there should be no discrimination against athletes based on their participation in testing or the results attained from a genetic or genomic test. A clear process, involving genetic counselling, should be outlined for dealing with unintentional discoveries that may confer a health risk to the participant.

Additionally, there is a capability gap in the genetic literacy of many current medical practitioners and the readiness of the medical industry to provide the relevant infrastructure to use genomics as part of usual care. Several experts have stated that the challenges for integration of clinical genomics into mainstream medical care include the education of providers and patients, the provisions of appropriate regulatory framework and the accrual of sufficient evidence to draw relevant conclusions [71]. Genetic information may be difficult to interpret for a sports medicine provider who has not had additional training in this area [72]. The risk conferred by gene variants differ significantly from the risk conferred from genetic tests for Mendelian or single-gene disorders. Genetic discoveries are often difficult to translate into clinical practice. There are complexities in determining which gene variants are associated with increased risk, which have no impact on exposure to risk and whether an increase in disease risk is clinically meaningful (e.g., from 15% to 16%). For this reason, it is important that practitioners understand the impact of the testing prescribed, and are able to provide counselling to the individual on the risks of genetic testing or to have access to a genetic counsellor should the need arise [71, 72].

### Unsafe use of technology e.g., CRISPR-Cas9 for genetic manipulation

The ease of accessing gene-editing techniques, such as the CRISPR-Cas 9 technique, may make this inexpensive, cut-and-paste type of gene-editing an attractive option for athletes wishing to use genetic performance enhancement. Sports performance is an area where current genetic knowledge already suggests somatic modifications that could provide performance enhancement [73]. Despite ease of access to this technology, gene-editing remains imprecise and should only be conducted by highly skilled specialists for specific medical indications and when approved by the appropriate authorities. The danger is that athletes and coaches are seen as potential early adopters of illicit performance enhancing technologies [74]. There is no role for gene-editing for the purposes of performance enhancement and all genetic manipulations are banned under the World Anti-Doping Agency Code [75]. Nevertheless, research efforts involving state-of-the-art ‘omics’ methods such as transcriptomics, proteomics and metabolomics are being intensified, also by FIMS and members of the Athlome Project Consortium, in order to identify robust molecular signatures of doping that have particular relevance in detecting gene-editing manipulations.

## The way forward

### International collaborations and utilising high throughput sequencing technologies to identify genes that contribute to exercise response

The challenge continues to be that it is difficult for one organisation to gather sufficiently high numbers of samples to provide meaningful analysis with clinical predictive value. The solution is large-scale multi-centre collaborations to drive research in this area and to ensure appropriate cohort size and homogeneity.

The cost of these rapidly improving sequencing techniques has been significantly reduced in the last decade. While the first human genome took $2.7 billion and almost 15 years to complete, the cost to sequence a genome has now been drastically reduced to close to $1000 (‘The $1000 genome’ era) [76]. The $1000 genome refers to an era of predictive and personalised medicine during which the cost of fully sequencing an individual’s genome is roughly USD $1000. This already allows scientists to sequence hundreds of thousands of genomes and will certainly have implications in advancing the field of exercise/injury genomics.

Two symposia were held in 2015 and 2016; the first in Greece and the second, in Slovenia, in partnership with the International Federation of Sports Medicine (FIMS), to review the main findings in exercise genetics and genomics and to explore promising trends and possibilities [14]. Among the participants, many were involved in ongoing collaborative studies (e.g., GAMES, Gene SMART, GENESIS and POWERGENE). A consensus emerged among participants that it would be advantageous to bring together all current studies and those recently launched into one new large collaborative initiative, which was subsequently named the Athlome Project Consortium. A website for the new consortium has been developed (www.athlomeconsortium.org), and clear goals have been established with steps to achieve these [14]. The intention is not for the Athlome Project Consortium to provide exclusivity but rather to serve as a model for sustainable and ethical research in sport and exercise medicine. In the few years since the launch of the Athlome project consortium, there has been much progress, with particular highlight the stimulation of multiple international collaborative research initiatives and joint research publications such as the present series published in BMC Genomics. The two previously mentioned GWAS investigations that involved members and cohorts associated with the Athlome consortium [55, 56] are further examples of encouraging outcomes to date. The main obstacle for further sustainable growth of the Athlome project consortium has been its operation during a time of sustained global research grant ‘famine’ for expensive large-scale collaborative research initiatives in sport and exercise medicine. More consortia and large multi-centre research initiatives are expected to follow such as the Genotype-Tissue Expression (GTEx) program (https://commonfund.nih.gov/gtex) recently launched by the NIH-USA and envisaged to provide valuable insights into the mechanisms of gene regulation by studying human gene expression and regulation in multiple tissues from healthy individuals; exploring disease-related perturbations in a variety of human diseases; and examining sexual dimorphisms in gene expression and regulation in multiple tissues.

### The usefulness of genetic testing in changing behavior

There are challenges that health and exercise professionals, along with coaches from high performance athletic programs, may face that relate to compliance with exercise or preventative health programs built on genetic analysis. Recent studies have demonstrated that direct-to-consumer cancer risk estimates do not appear to affect health-related behaviors positively or negatively [77]. Despite being informed of their relative risk for a variety of cancers, most adults did not significantly change their diet, exercise, advanced care planning, or cancer screening behaviors [78]. One must ask; if adults are unlikely to change their health behavior in relation to a serious medical condition such as cancer, will personalised genomics be relevant in the exercise and sports-injury space? Understanding of the various behaviors that relate to sports injury risk and exercise prescription is needed before the relevance of genetic testing in these fields can be uniformly applied. Many studies have investigated the compliance or adherence to health-related exercise programs and interventions in relation to sports injury prevention [79–82]. In relation to elite sport, a study involving top UEFA football elite clubs demonstrated that athletes’ adherence to the injury prevention programs remain varied, although coach compliance was rated as ‘high’ [83]. This implies that attitudes towards intervention programs reflect the beliefs of the individual and not necessarily that of coaches or support staff. Collard et al. recommend that a behavioral approach and ‘intervention mapping’ is required when defining a sports-injury prevention program to increase the risk of adherence [84]. A systematic approach is required in planning health promotion strategies, under which injury prevention and exercise prescription may be considered [85]. In addition, any possibility of introducing genetic testing in the elite sport scenario would need to be accompanied by education to athletes and support staff in order to improve genetic literacy.

## Conclusions

Having considered the current level of scientific knowledge, the opinion of the stakeholders of this joint statement is that the predictive value of such tests is too low to warrant clinical application. The risks associated with this type of testing, including privacy issues, unintended genetic discoveries and erroneous advice based on poor evidence, should be mitigated with understanding of the test limitations, management of the data produced, and avoidance of advice that is not supported by scientific evidence. Additionally, organisations interested in the concept of genetic testing for exercise prescription and injury prevention should develop and clearly articulate the ethical framework within which that organisation is prepared to conduct genetic testing and research. Genetic testing for the purposes of exercise prescription and injury prevention may in the future be a legitimate and valid use of genetic information contributing health benefits to individuals with a wide range of athletic ability and injury predisposition. In order for genetic testing to become a useful component of medical practice, intensive international collaboration will be required. 
